# A ketogenic diet reduces hepatic alcohol metabolism and alcohol consumption in rats

**DOI:** 10.1038/s41386-026-02383-5

**Published:** 2026-03-20

**Authors:** Sophie K. Elvig, Adrienne McGinn, Xinyi Li, Janaina C. M. Vendruscolo, Juan L. Gomez, Robert Pawlosky, Bryan Mackowiak, Luis Gonzalez, M. Todd King, Michael Michaelides, Bin Gao, Nora D. Volkow, George F. Koob, Corinde E. Wiers, Leandro F. Vendruscolo

**Affiliations:** 1 Neurobiology of Addiction Section, Integrative Neuroscience Research Branch, National Institute on Drug Abuse, Intramural Research Program, National Institutes of Health, Baltimore, MD USA; 2 Department of Psychiatry, University of Pennsylvania, Perelman School of Medicine, Philadelphia, PA USA; 3 Biobehavioral Imaging and Molecular Neuropsychopharmacology Section, Neuroimaging Research Branch, National Institute on Drug Abuse, Intramural Research Program, National Institutes of Health, Baltimore, MD USA; 4 Office of the Scientific Director, Division of Intramural Clinical and Biological Research, National Institute on Alcohol Abuse and Alcoholism, National Institutes of Health, Bethesda, MD USA; 5 Laboratory of Liver Diseases, Division of Intramural Clinical and Biological Research, National Institute on Alcohol Abuse and Alcoholism, National Institutes of Health, Bethesda, MD USA; 6 Laboratory of Neuroimaging, Division of Intramural Clinical and Biological Research, National Institute on Alcohol Abuse and Alcoholism, National Institutes of Health, Bethesda, MD USA; 7 Stress and Addiction Neuroscience Unit, Intramural Research Program, National Institute on Drug Abuse. National Institutes of Health, Baltimore, MD USA; 8 Division of Intramural Clinical and Biological Research, National Institute on Alcohol Abuse and Alcoholism, National Institutes of Health, Bethesda, MD USA; 9 Present Address: Department of Pharmacology and Physiology, School of Medicine, University of Maryland, Baltimore, MD USA

**Keywords:** Reward, Metalloproteins

## Abstract

Previous work showed that rats that were exposed to a high-fat, low-carbohydrate/protein ketogenic diet (KD) exhibited elevated blood alcohol levels following alcohol exposure compared with rats fed regular chow. Additionally, the administration of a KD prior to alcohol exposure (i.e., a history of KD) reduced alcohol consumption in alcohol-dependent rats that were no longer on the diet. In the present study, we investigated the mechanisms by which a KD alters alcohol metabolism and tested whether ongoing KD exposure reduces alcohol consumption in rats. We hypothesized that chronic KD exposure alters hepatic alcohol-metabolizing enzymes, slows alcohol metabolism, and reduces alcohol self-administration in alcohol-dependent rats. We found that male and female rats maintained on a KD had higher blood alcohol levels, lower hepatic alcohol dehydrogenase 1 protein levels, and a higher nicotinamide adenine dinucleotide [NAD+]/[NADH] ratio in the liver cytoplasm compared with chow-fed control rats. Furthermore, KD-fed rats demonstrated lower brain glucose uptake relative to chow-fed control rats. In a model of alcohol dependence, the KD reduced alcohol consumption in male, but not female, rats compared with chow-fed rats. These findings suggest that a KD alters brain energetics and alcohol metabolism, which may contribute to reduced alcohol consumption in male rats.

## Introduction

Excessive alcohol drinking has detrimental health consequences, including an increased risk of developing liver cirrhosis and alcohol use disorder (AUD). A comparative risk assessment study estimated that 5% of deaths and disability-adjusted life-years globally are attributable to alcohol [1]. Identifying factors that modulate alcohol metabolism and excessive drinking has important implications for reducing alcohol-related pathology. Among these factors, diet has recently emerged as a potential modulator [2].

A ketogenic diet (KD) is a high-fat, low-carbohydrate, low-protein diet that promotes endogenous production of ketone bodies (i.e., acetoacetate, acetone, and β-hydroxybutyrate) through fat breakdown [3]. In the state of nutritional ketosis, which also occurs during fasting, ketone bodies serve as an alternative energy source for the body and brain when glucose availability is reduced [3]. Because acute and chronic alcohol exposure reduces brain glucose metabolism [2, 4, 5], we and others have begun to study the effects of targeting energy metabolism with KD on alcohol metabolism, withdrawal, and consumption in preclinical and clinical studies [2, 6, 7].

Despite evidence supporting the therapeutic potential of KD for AUD, previous work showed that male rats maintained on a KD exhibited markedly higher blood alcohol levels (BALs) following alcohol exposure compared with chow-fed controls [8], raising concerns about heightened alcohol intoxication during relapse in individuals with AUD adhering to a KD. Alcohol is metabolized in the liver through sequential oxidation to acetaldehyde and acetate by alcohol dehydrogenase (ADH) and acetaldehyde dehydrogenase (ALDH), respectively [9], a process that requires the cofactor nicotinamide adenine dinucleotide (NAD^+^) to accept a hydrogen and form NADH [10]. KD induces a metabolic state resembling fasting, which has been associated with decreased ADH activity and slower alcohol elimination [11, 12]. Similarly, low carbohydrate intake, which is characteristics of KD, has been linked to impaired alcohol metabolism [13, 14]. However, no prior study has directly examined the effects of KD on hepatic ADH, ALDH, or the [NAD^+^]/[NADH] ratio.

Clinical and preclinical studies suggest that a KD may reduce alcohol withdrawal and craving. In inpatients with AUD undergoing alcohol detoxification, KD reduced the need for benzodiazepine medication, withdrawal symptoms, and craving [8], and rodent studies showed similar reductions in alcohol withdrawal severity [7]. KD-fed, non-alcohol-dependent mice self-administered less alcohol than chow-fed controls [15], and KD prevented social stress-induced alcohol drinking in non-alcohol-dependent female mice [16]. We previously showed that a history of KD blocked the escalation of alcohol drinking caused by alcohol vapor-induced dependence in rats [8]. However, attempts to measure self-administration during KD exposure were confounded by large differences in BALs during alcohol vapor exposure. Thus, it remains unknown whether ongoing KD exposure during dependence alters alcohol consumption during acute withdrawal.

In the present study, we evaluated the impact of chronic KD exposure on BALs, hepatic enzymes, and cofactors involved in alcohol metabolism. We also examined whether ongoing KD exposure affects operant alcohol self-administration during alcohol withdrawal in alcohol-dependent rats. To control for differences in alcohol metabolism, KD-fed rats were exposed to lower concentrations of alcohol vapor to achieve BALs comparable to chow-fed rats. In the alcohol vapor model of dependence, rats were passively exposed to alcohol vapor for 14 h daily to achieve BALs of ~200 mg/dl. During the 10 h of daily withdrawal from alcohol vapor, rats were tested for operant oral alcohol self-administration, typically 4–8 h into withdrawal. This model reliably produces somatic signs of withdrawal (e.g., tremors) and motivational signs of withdrawal (e.g., elevated reward thresholds and escalated alcohol drinking) [17]. We hypothesized that a KD would reduce alcohol metabolism by lowering hepatic alcohol dehydrogenase 1 (ADH1) protein levels, and altering the [NAD^+^]/[NADH] ratio, and reducing alcohol consumption in dependent rats.

## Materials and methods

### Animals

Adult male and female Wistar rats (Charles River, Raleigh, NC, USA), at least 8 weeks of age at the start of the experiments, were used. Rats were group-housed 2–3 per cage in a temperature-controlled (21 °C ± 2 °C) vivarium on a reverse 12 h/12 h light/dark cycle (lights on at 8:00 PM), with *ad libitum* access to food and water throughout the study, except during the 30-min alcohol drinking sessions when food was unavailable. All animal procedures adhered to the National Institutes of Health Guide for the Care and Use of Laboratory Animals and were approved by the Animal Care and Use Committee of the National Institute on Drug Abuse, Intramural Research Program.

### Diet

All rats were initially maintained on a standard chow diet (2018 Teklad Rodent Diet, INOTIV, Indianapolis, IN, USA) and subsequently allocated to two groups: one group remained on chow, and the other received a high-fat, low-carbohydrate, low-protein KD (catalog no. F3666, Bio-Serv, Flemington, NJ, USA) for the duration of the experiment. The KD contained 93% of calories from fat, 2% from carbohydrates, and 5% from protein. The chow diet contained 13% of calories from fat, 57% from carbohydrates, and 30% from protein.

### Blood ketone, glucose and alcohol measurements

Male (*n* = 8) and female (*n* = 8) rats were fed their assigned diet for 8 weeks, and ketone and glucose levels were measured at weeks 1, 2, 4, 6, and 8. For these measurements, rats were not exposed to alcohol or fasting. Blood was collected by tail clipping 2–6 h into the dark cycle. Blood ketone levels were measured using a Precision Xtra Blood Ketone Monitoring System (Abbott, Alameda, CA, USA), and blood glucose levels were measured using a glucometer (Tyson Bioresearch, Zhunan, Taiwan).

Blood was also collected by tail clipping from the same rats during the dark cycle following 4 h of alcohol vapor exposure at weeks 1, 2, 4, 6, and 8. This short-term alcohol exposure does not produce alcohol dependence. BALs were measured using an alcohol analyzer (Analox Technologies North America, Toronto, Ontario, Canada).

### Positron emission tomography for [18F]fluorodeoxyglucose brain uptake

To assess the effects of diet on brain glucose metabolism, we performed small-animal positron emission tomography (PET) to compare [^18^F]fluorodeoxyglucose ([^18^F]FDG) whole-brain uptake in a separate cohort of alcohol-naïve male rats exposed to 8 weeks of KD or chow (*n* = 4 per group). PET scans were obtained following an overnight fast, during which water was available *ad libitum*. Before the scan, the rats were brought to a holding vivarium. Rats were weighed, and [^18^F]FDG was prepared in a zero-dead space syringe for a target dose of 1 µCi/g body weight. Rats were injected intraperitoneally with [^18^F]FDG and allowed 30 min for uptake in home cages. Rats were then anesthetized with isoflurane (5% isoflurane/oxygen mixture), placed on the scanner bed, and maintained under sedation with a 1–2% isoflurane/oxygen mixture for the remainder of the scan. Imaging was performed using a nanoScan PET/computed tomography (CT) scanner (Mediso, Arlington, VA, USA): PET for 20 min followed by CT for 3 min. After scanning, rats were given food and returned to the holding vivarium for at least 24 h to allow for radiotracer decay. Experiments were conducted during the dark cycle.

### Liver ADH1 protein, lactate, pyruvate, NAD+, and NADH measurements

A separate cohort of alcohol-naïve male (*n* = 8) and female (*n* = 8) rats were euthanized after 8 weeks of KD or chow exposure. Liver samples were collected and snap-frozen in dry ice-cold isopentane, then stored at −80 °C until use in the assays described below.

### Gas chromatography-mass spectrometry analysis of pyruvate, lactate, free cytosolic [NAD^+^]/[NADH]

The labeled internal standards ^13^C_3_-sodium pyruvate and ^13^C_3_-lactatic acid (Cambridge Isotope Labs, Cambridge, MA, USA) were used for the quantification of lactate and pyruvate. Approximately 20 mg of frozen liver tissue was added to 80 µl of 3% perchloric acid solution in conical tubes with 8–10 glass beads and homogenized on a laboratory Mini Beadbeater (Biospec Products, Bartlesville OK, USA) at 5000 beats/min according to previously published procedures [18]. Samples were kept on ice and centrifuged at 4 °C for 3 min in a Sorvall centrifuge (rotor, FA-45-30-11) at 2000 rpm, forming a pelleted residue. Fifty microliters of supernatant were neutralized with 8 µl of a KCO_3_ (3 M) solution in conical snap tubes. The neutralized solution was again centrifuged as described above, and the ^13^C-labeled internal standards were added in approximately two-fold excess of the concentrations of the individual analytes in tissue homogenates. Ten microliters of the supernatant were dried under nitrogen (99.99%) and reacted with 5 µl of *N*-methyl-*N*-(tert-butylmethylsilyl) trifluoroacetamide with 1% tert-butyldimethylchlorosilane reagent (Thermo Scientific, Rockford, IL, USA) and 5 µl acetonitrile at 90 °C for 5 min to form tertiary butyldimethylsilyl esters [18].

Samples were analyzed on an Agilent 5973 quadrupole gas chromatograph/mass spectrometer (Agilent, Wilmington, DE, USA). One microliter of sample solution was injected onto a 250 μm × 30 m capillary DB-1 column (Agilent, Wilmington, DE, USA) in splitless injection mode using helium as the carrier gas. The injector was set to 270 °C and the transfer line to 280 °C. Oven temperature was programmed from 80 °C to 325 °C at 15 °C/min. The mass spectrometer operated in electron impact mode (70 eV), and the quadrupole mass analyzer scanned for ions that corresponded to a loss of 15 mass units (-CH_3_) from the molecular ion and the base peak of each analyte and its corresponding ^13^C-labeled internal standard using selected ion monitoring.

Free [NAD^+^]/[NADH] was calculated from ratios of concentrations of pyruvate to lactate using the equilibrium reaction of lactate dehydrogenase (LDH) with the equilibrium constant equal to 1.111 × 10^−4^ [19].

Free [NAD^+^]/[NADH] - cytosolic [20]

$$\frac{\left[{{Pyr}}^{-}\right]}{\left[{{Lac}}^{-}\right]}\times \frac{[{NADH}]}{\left[{{NAD}}^{+}\right]}={K}_{{LDH}}=1.11\times {10}^{-4}$$

Rearranged

$$\frac{\left[{{NAD}}^{+}\right]}{\left[{NADH}\right]}=\frac{\left[{{Pyr}}^{-}\right]}{[{{Lac}}^{-}]}\times \frac{1}{{K}_{{LDH}}}$$

### Western blot analysis of liver ADH1 protein

Liver tissue was homogenized in radioimmunoprecipitation assay buffer (Cell Signaling Technology, Danvers, MA, USA) that contained a protease/phosphatase inhibitor cocktail (Cell Signaling Technology, Danvers, MA, USA) using a bead homogenizer. The homogenate was centrifuged at 10,000 × *g* for 10 min at 4 °C. After homogenization, protein concentration was determined by the bicinchoninic acid (BCA) assay (Pierce BCA protein assay, ThermoFisher Scientific, Waltham, MA, USA). The supernatants were mixed with loading buffer, and 20 µg of protein for each sample was separated by sodium dodecyl sulfate-polyacrylamide gel electrophoresis. The samples then underwent electrophoretic transfer to nitrocellulose membranes (ThermoFisher Scientific, Waltham, MA, USA). The membranes were blocked in 5% milk, incubated with ADH1 primary antibody (1:1000, catalog no. 5295, Cell Signaling Technology, Danvers, MA, USA) overnight in Tris-buffered saline with Tween 20 (TBST) that contained 5% bovine serum albumin, washed with TBST, and incubated with species-specific peroxidase-conjugated secondary antibody (1:5000, Cell Signaling Technology, Danvers, MA, USA) for 1 h. Protein bands were visualized by SuperSignal West Pico (Thermo Fisher Scientific, Waltham, MA, USA). Protein densitometry values were normalized to the loading control protein α-tubulin (1:1000, catalog no. 3873S, Cell Signaling Technology, Danvers, MA, USA). Immunoreactivity was quantified using ImageJ software (National Institutes of Health, Bethesda, MD, USA).

### Operant alcohol self-administration during withdrawal from alcohol vapor

Different cohorts of male (*n* = 20) and female (*n* = 31) rats were trained to orally self-administer alcohol (10% w/v) in standard operant conditioning chambers (Med Associates, St. Albans, VT, USA) under a fixed-ratio 1 schedule of reinforcement, in which an operant response on the designated alcohol lever or water lever was reinforced with 0.1 ml of solution as previously described [21]. After the acquisition of operant alcohol self-administration in 8–12 30-min sessions, the rats were assigned to the KD or chow diet, which was maintained for the duration of the experiment. The rats were fed their assigned diet for 5 days before starting alcohol vapor exposure. The rats were exposed daily to chronic, intermittent alcohol vapor exposure (14 h on and 10 h off daily) to produce somatic and motivational signs of alcohol dependence [17]. Rats on the KD and chow diet were housed in independent alcohol vapor chambers with different alcohol concentrations to achieve similar BALs in both groups. We designed this “clamping” method to consider differences in alcohol metabolism between the KD and chow groups [8]. The alcohol-delivering pump stroke rate was adjusted to reduce the quantity of alcohol that was vaporized during exposure in the KD group. For example, the pump was adjusted to deliver 14–20 drops of alcohol per minute into the vaporizing flask for chow-fed rats, whereas ~40% fewer deliveries were used for KD-fed rats. Each drop delivered 9 ml of 95% ethyl alcohol.

Thirty-minute operant alcohol self-administration sessions continued 2–3 times weekly 6–8 h into alcohol withdrawal. This intermittent exposure reliably produces escalation of alcohol intake during both acute and protracted abstinence [22]. Experiments were conducted during the dark cycle.

### Statistical analysis

Blood glucose levels, blood ketone levels, body weights, BALs, and alcohol self-administration data were analyzed using three-way repeated-measures analysis of variance (ANOVA), with time or operant sessions as within-subjects factors and diet (KD *vs*. chow diet) and sex (male *vs*. female) as the between-subjects factors. Liver measurements and average alcohol self-administration data were analyzed using two-way ANOVAs, with diet (KD *vs*. chow diet) and sex (male *vs*. female) as between-subjects factors. *Post hoc* comparisons were performed using the Duncan’s test. The data are expressed as the mean ± standard error of the mean (SEM). Pearson’s correlation analyses were conducted separately for each sex and diet to identify group-specific differences in correlation patterns. Prism 8 software (GraphPad, San Diego, CA, USA) was used for figure preparation. Statistica 13 software (TIBCO Software, Palo Alto, CA, USA) was used for statistical analyses.

For PET analysis, scans were reconstructed on using Mediso scanner software. Scans were co-registered with PMOD (Zürich, Switzerland) to a magnetic resonance imaging template to ensure all scans were at the same coordinate space. Statistical Parametric Mapping (SPM12, London, UK) was used to compare [^18^F]FDG uptake between groups using Student’s *t* tests for voxel-wise analysis. Cluster plots showed differences between the KD and chow diet groups.

The accepted level of significance for all tests was *p* ≤ 0.05.

## Results

### Effect of a KD on blood glucose, ketone, and alcohol levels

**Fig. 1:**
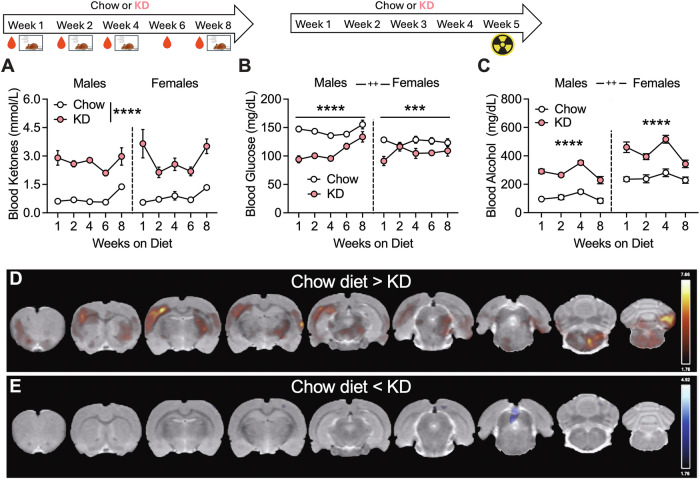
Blood ketone, glucose, and alcohol levels and brain [^18^F]fluorodeoxyglucose uptake in rats that were maintained on a ketogenic diet (KD) or chow diet for 8 weeks. The top-left schematic shows the timeline of the biochemical measurement experiment. **A** Higher blood ketone levels, **B** lower blood glucose levels, and **C** Higher blood alcohol levels in rats that were maintained on the KD compared with the chow diet. *n* = 8 male and female rats per group. ***p < 0.001, ****p < 0.0001, difference between chow and KD; ^++^*p* < 0.01, difference between males and females. The top-right schematic shows the timeline of the positron emission tomography (PET) experiment. **D** The chow group exhibited greater [^18^F]FDG uptake in multiple cortical and subcortical regions and hindbrain regions compared with the KD group. **E** Compared with the chow diet, rats in the KD group exhibited greater [^18^F]FDG uptake in only one region. *n* = 4 male rats per group. Higher [^18^F]FDG uptake in the chow group *vs*. the KD group is shown in red, and lower [^18^F]FDG uptake is shown in blue (*p* < 0.05).

Male and female KD-fed rats exhibited higher blood ketone levels than chow-fed rats across the 8-week period (Fig. 1A; diet effect: *F*_1,28_ = 104.91, *p* < 0.0001; sex effect: *F*_1,28_ = 0.32, *p* = 0.5743; diet × sex interaction: *F*_1,28_ = 0.03, *p* = 0.8545; time effect: *F*_4,112_ = 11.25, *p* < 0.0001; diet × time interaction: *F*_4,112_ = 4.50, *p* = 0.0021; sex × time interaction: *F*_4,112_ = 0.99, *p* = 0.4150; diet × sex × time interaction: *F*_4,112_ = 2.00, *p* = 0.0988).

Blood glucose levels were lower in KD-fed rats compared with chow-fed rats, and male rats exhibited higher glucose levels than females. The glucose-lowering effect of the KD was more pronounced in males than in females (Fig. 1B; diet effect: *F*_1,28_ = 52.42, *p* < 0.0001; sex effect: *F*_1,28_ = 8.36, *p* = 0.0073; diet × sex interaction: *F*_1,28_ = 4.58, *p* = 0.0413; time effect: *F*_4,112_ = 5.33, *p* = 0.0006; diet × time interaction: *F*_4,112_ = 4.51, *p* = 0.0020; sex × time interaction: *F*_4,112_ = 4.34, *p* = 0.0027; diet × sex × time interaction: *F*_4,112_ = 2.28, *p* = 0.0655). *Post hoc* comparisons of the diet × sex interaction indicated that both KD-fed males (*p* < 0.0001) and females (*p* = 0.0016) had lower glucose levels than their respective chow-fed controls.

BALs were higher in KD-fed rats compared with chow-fed rats, and females exhibited higher BALs than males (Fig. 1C; diet effect: *F*_1,28_ = 124.65, *p* < 0.0001; sex effect: *F*_1,28_ = 77.32, *p* = 0.0073; diet × sex interaction: *F*_1,28_ = 0.05, *p* = 0.8185; time effect: *F*_3,84_ = 26.67, *p* < 0.0001; diet × time interaction: *F*_3,84_ = 6.64, *p* = 0.0004; sex × time interaction: *F*_3,84_ = 0.61, *p* = 0.6110; diet × sex × time interaction: *F*_3,84_ = 0.78, *p* = 0.5079).

### Effect of a KD on [^18^F]FDG brain uptake

**Fig. 2:**
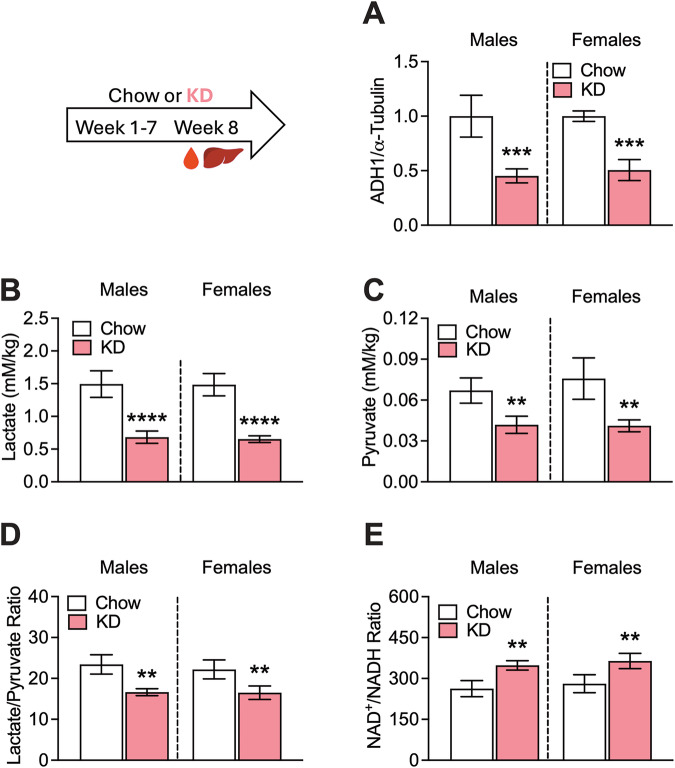
Liver alcohol dehydrogenase 1 (ADH1) protein, cytosolic lactate, and pyruvate levels, and lactate/pyruvate and [NAD^+^]/[NADH] ratios in rats that were maintained on a ketogenic diet (KD) or chow diet for 8 weeks. The top-left schematic shows the timeline of the experiment. Compared with chow-fed rats, KD-fed rats exhibited **A** lower ADH1 protein levels, **B** lower lactate levels, **C** lower pyruvate levels, **D** lower lactate/pyruvate ratio, and **E** higher [NAD^+^]/[NADH] ratio. **p < 0.01, ***p < 0.001, ****p < 0.0001, difference between chow and KD. *n* = 8 per group, except for ADH1 i*n* male KD-fed rats (*n* = 6).

Brain [^18^F]FDG uptake was assessed in male rats only. Figure 2A, B shows whole-brain [^18^F]FDG uptake in rats maintained on chow versus a KD. Chow-fed rats (Fig. 2A, red) exhibited greater [^18^F]FDG uptake across multiple cortical, subcortical, and hindbrain regions compared with KD-fed rats. In contrast, KD-fed rats exhibited greater [^18^F]FDG uptake in only a single brain region relative to chow-fed rats (Fig. 2B, blue).

### Effect of a KD on body weight

Body weight was lower in male KD-fed rats compared with male chow-fed rats, whereas no diet-related differences were observed in females (Supplementary Fig. S1).

### Effect of a ketogenic diet on liver enzymes and metabolites

In this cohort of alcohol-naïve rats, KD-fed males and females exhibited higher blood ketone levels and lower blood glucose levels compared with chow-fed rats, along with reduced body weight in KD-fed males (Supplementary Fig. S2A–C).

Liver ADH1 protein levels (Fig. 2A; diet effect: *F*_1,26_ = 18.38, *p* = 0.0002; sex effect: *F*_1,26_ = 0.05, *p* = 0.8283; diet × sex interaction: *F*_1,26_ = 0.05, *p* = 0.8283), lactate levels (Fig. 2B; diet effect: *F*_1,28_ = 33.07, *p* < 0.0001; sex effect: *F*_1,28_ = 0.02, *p* = 0.8931; diet × sex interaction: *F*_1,28_ = 0.01, *p* = 0.9413), pyruvate levels (Fig. 2C; diet effect: *F*_1,28_ = 9.61, *p* = 0.0044; sex effect: *F*_1,28_ = 0.17, *p* = 0.6826; diet × sex interaction: *F*_1,28_ = 0.24, *p* = 0.6274), and the lactate/pyruvate ratio (Fig. 2D; diet effect: *F*_1,28_ = 10.79, *p* = 0.0027; sex effect: *F*_1,28_ = 0.13, *p* = 0.7201; diet × sex interaction: *F*_1,28_ = 0.08, *p* = 0.7743) were all lower in KD-fed rats compared with chow-fed rats, regardless of sex. The liver cytoplasmic [NAD^+^]/[NADH] ratio was higher in KD-fed rats than in chow-fed rats (Fig. 2E; diet effect: *F*_1,28_ = 9.41, *p* = 0.0047) with no effects of sex or diet x sex interactions (sex effect: *F*_1,28_ = 0.3754, *p* = 0.5450; diet × sex interaction: *F*_1,28_ = 0.0012, *p* = 0.9732).

Correlation analyses revealed diet-and sex-specific relationships. In chow-fed males (Supplementary Table S1), glucose levels correlated positively with the lactate/pyruvate ratio (*p* < 0.01) and negatively with the [NAD^+^]/[NADH] ratio (*p* < 0.01), while body weight correlated negatively with ADH1 levels (*p* < 0.05). ADH1 levels correlated negatively with lactate and pyruvate levels (*p* < 0.05), lactate levels correlated positively with pyruvate levels (*p* < 0.05), and the lactate/pyruvate ratio correlated negatively with the [NAD^+^]/[NADH] ratio (*p* < 0.0001).

In KD-fed males (Supplementary Table S2), glucose levels correlated negatively with ketone levels (*p* < 0.05), lactate levels correlated positively with pyruvate levels (*p* < 0.001), and the lactate/pyruvate ratio correlated negatively with the [NAD^+^]/[NADH] ratio (*p* < 0.0001).

Similar correlations between the lactate/pyruvate ratio and [NAD^+^]/[NADH] were observed in both chow- and KD-fed females (Supplementary Table S3, 4).

### Effect of a KD on alcohol consumption in alcohol-dependent rats

To achieve comparable BALs between diet groups, rats were housed in separate alcohol vapor chambers that delivered lower concentrations of alcohol vapor to KD-fed than to chow-fed rats. BAL measurements confirmed equivalent BALs in the KD and chow groups across the 7–9 weeks of vapor exposure (Supplemental Fig. S2).

**Fig. 3:**
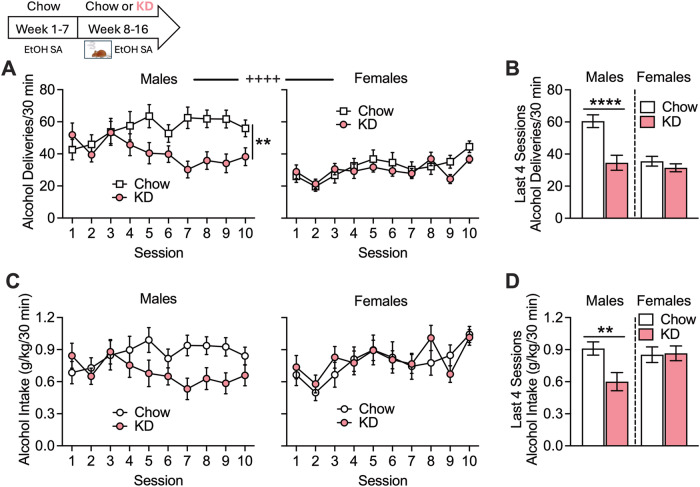
Operant oral alcohol self-administration that occurred during withdrawal from daily alcohol vapor exposure in alcohol-dependent rats that were maintained on a ketogenic diet (KD) or chow diet for 7–9 weeks. The top-left schematic shows the timeline of the biochemical measurement experiment. Compared with chow-fed rats, male KD-fed rats but not female rats exhibited (**A**, **B**) fewer lever presses for alcohol and (**C**, **D**) alcohol intake. Note that the apparent sex differences in the number of lever presses and alcohol intake in g/kg are due to large sex differences in body weight. **p < 0.01, ****p < 0.0001, difference between chow and KD. Males: *n* = 10 per group. Females: *n* = 15 for KD and *n* = 16 for chow.

Despite equivalent BALs, male KD-fed rats exhibited fewer lever presses for alcohol during operant self-administration sessions conducted during withdrawal compared with chow-fed males (Fig. 3A). Overall, operant responding was higher in males than females (diet effect: *F*_1,47_ = 7.29, *p* = 0.0096; sex effect: *F*_1,47_ = 30.75, *p* < 0.0001; diet × sex interaction: *F*_1,47_ = 4.04, *p* = 0.0502; time effect: *F*_9,423_ = 2.79, *p* = 0.0035; time × diet interaction: *F*_9,423_ = 3.69, *p* = 0.0002; time × sex interaction: *F*_9,423_ = 1.45, *p* = 0.1651; time × diet × sex interaction: *F*_9,423_ = 1.80, *p* = 0.0656). *Post hoc* comparisons confirmed reduced responding in KD-fed males (*p* = 0.0018), with no diet effects in females. We calculated the average number of alcohol deliveries in the last four self-administration sessions (Fig. 3B) when BALs were highest in both males and females (Supplementary Fig. S3A, B). Male KD-fed rats made fewer lever presses than male chow-fed rats, and male rats made more lever presses than female rats (diet effect: *F*_1,47_ = 18.76, *p* < 0.0001; sex effect: *F*_1,47_ = 16.44, *p* = 0.0002; diet × sex interaction: *F*_1,47_ = 9.91, *p* = 0.0029; *post hoc* test: *p* < 0.0001).

Alcohol intake expressed as g/kg (Fig. 3C) did not differ by diet or sex across sessions (diet effect: *F*_1,47_ = 1.00, *p* = 0.3228; sex effect: *F*_1,47_ = 0.08, *p* = 0.7830; diet × sex interaction: *F*_1,47_ = 2.03, *p* = 0.1607; time effect: *F*_9,423_ = 3.23, *p* = 0.0008; time × diet interaction: *F*_9,423_ = 1.89, *p* = 0.0518; time × sex interaction: *F*_9,423_ = 2.02, *p* = 0.0360; time × diet × sex interaction: *F*_9,423_ = 1.04, *p* = 0.4099). However, for the average of the last four self-administration sessions, male KD-fed rats exhibited lower alcohol intake (in g/kg) than male chow-fed rats, but the KD did not affect alcohol intake in females (diet effect: *F*_1,47_ = 3.84, *p* = 0.0559; sex effect: *F*_1,47_ = 1.87, *p* = 0.1781; diet × sex interaction: *F*_1,47_ = 5.54, *p* = 0.0384; Duncan’s *post hoc* test, *p* = 0.0096). Note that the apparent sex differences in the number of lever presses and alcohol intake in g/kg are due to large sex differences in body weight.

For lever presses for water (Supplementary Fig. S3A,B), male rats made more presses than female rats (sex effect: *F*_1_, _47_ = 36.60, *p* < 0.0001) and the number of presses varied across sessions (time effect: *F*_9_, _423_ = 3.33, *p* = 0.0006; sex × time interaction: *F*_9, 423_ = 4.09, *p* < 0.0001) but was unaffected by the diet (diet effect: *F*_1, 47_ = 0.33, *p* = 0.5683; diet × sex interaction: *F*_1, 47_ = 0.02, *p* = 0.9011; diet × time interaction: *F*_9, 423_ = 0.58, *p* = 0.8173; diet × sex × time interaction: *F*_9, 423_ = 0.76, *p* = 0.6494).

For crossovers (Supplementary Fig. S3C, D), KD-fed rats exhibited fewer crossovers than chow-fed rats (diet effect: *F*_1, 39_ = 4.57, *p* = 0.0388) regardless of sex (sex effect: *F*_1, 39_ = 0.01, *p* = 0.9923; diet × sex interaction: *F*_1, 39_ = 0.05, *p* = 0.8313) and crossovers varied across sessions (time effect: *F*_9, 351_ = 2.98, *p* = 0.0020; diet × time interaction: *F*_9, 351_ = 0.63, *p* = 0.7747; sex × time interaction: *F*_9, 351_ = 3.06, *p* = 0.0015; diet × sex × time interaction: *F*_9, 351_ = 1.09, *p* = 0.3682).

## Discussion

In the present study, we found that male and female KD-fed rats exhibited lower blood glucose levels and higher ketone levels and BALs following alcohol vapor exposure compared with chow-fed rats. The KD reduced hepatic ADH1 protein expression and levels of pyruvate, and lactate, while increasing the [NAD^+^]/[NADH] ratio. Exploratory PET imaging in males revealed that KD exposure also reduced whole-brain glucose metabolism, suggesting a shift toward ketone-based energy production. We observed sex-specific effects of the KD on body weight and alcohol self-administration (i.e., the number of operant responses and intake in g/kg). The KD reduced these measures in males but not in females. Altogether, these results suggest that ketosis reduces alcohol metabolism via changes in liver enzymes and redox cofactors in both sexes and reduces alcohol consumption in a sex-dependent manner.

As expected and consistent with previous studies [8, 23, 24], the KD increased blood ketone levels and decreased blood glucose levels, confirming the induction of nutritional ketosis. In clinical and preclinical neuroimaging studies, ketosis has been associated with decreased brain glucose metabolism and increased brain acetoacetate and β-hydroxybutyrate metabolism [25–28]. Similar metabolic shifts have been reported in individuals with AUD during intoxication and early abstinence compared with occasional drinkers [4, 5, 29]. This energetic reprogramming may contribute to alterations in alcohol metabolism and alcohol self-administration.

Our findings provide new insights into the metabolic and behavioral effects of a KD related to alcohol exposure. Consistent with our previous studies in male rats [8], acute 4-h alcohol vapor exposure led to higher BALs in male and female KD-fed rats compared with chow-fed controls, suggesting a lower hepatic capacity for alcohol oxidation during ketosis. A KD mimics a state of fasting, and fasting has been shown to reduce alcohol metabolism [10, 12, 30]. Alcohol is largely metabolized in the liver through sequential oxidation to acetaldehyde by ADH1 and then to acetate by ALDH [9]. The lower hepatic ADH1 protein levels observed in the present study may explain this effect. ADH1 is essential for converting alcohol to acetaldehyde and is a rate-limiting step in alcohol metabolism. Rodents lacking a functional *Adh1* gene and rodents exposed to pharmacological ADH1 inhibition exhibited decreased alcohol elimination rates [31, 32]. However, ADH1 protein expression alone cannot conclusively determine the rate of hepatic alcohol oxidation. Additional measures, such as direct ADH enzymatic activity assays and assessments of other key alcohol-metabolizing pathways, including ALDH and CYP2E1, would be required to fully characterize the impact of a KD on hepatic alcohol metabolism. Although our findings provide initial evidence that a KD may impair hepatic alcohol clearance, the specific enzymatic mechanisms underlying this effect remain to be clarified.

The liver is a critical metabolic hub in maintaining energy homeostasis. To our knowledge, no previous studies have systematically examined net effects of a KD on the hepatic [NAD^+^]/[NADH] redox ratio. In the present study, KD-fed rats of both sexes exhibited lower cytosolic levels of lactate and pyruvate, a lower lactate/pyruvate ratio, and a higher [NAD^+^]/[NADH] redox ratio. During glycolysis, glucose is metabolized to pyruvate in the cytosol, a process that requires the reduction of NAD^+^ to NADH. Pyruvate may subsequently be converted to lactate in the cytosol or transported into mitochondria to fuel the tricarboxylic acid cycle. The observed reductions in pyruvate and lactate levels are consistent with a metabolic shift away from glycolysis under KD conditions, resulting in reduced pyruvate and lactate production and increased NAD^+^ levels [33]. Supporting the conservation of NAD^+^ hypothesis, the addition of glucose decreased the cytosolic [NAD^+^]/[NADH] ratio, an effect that was blunted by pharmacological inhibition of glycolysis and by inclusion of fatty acids and β-hydroxybutyrate in the incubation medium [34]. Furthermore, neuroimaging studies indicated that exogenous ketones increased brain NAD^+^ levels and the [NAD^+^]/[NADH] ratio [35]. Changes in liver NAD^+^ levels may affect ADH activity, which requires NAD^+^ in the initial step of alcohol oxidation to acetaldehyde in the cytosol [10]. Accordingly, compounds such as formamides (e.g., isopropylformamide, cyclohexylformamide, and 3-butylthiolane 1-oxide) that bind to the enzyme-NADH complex inhibit alcohol metabolism [36]. In rodents, the administration of NAD precursors prior to alcohol exposure increased NAD^+^ levels in liver (as well as in blood, brain, fat and kidney) and reduced BALs [37]. However, one limitation of the present study is our inability to distinguish between cytosolic and mitochondrial NAD^+^/NADH pools. These compartments support distinct metabolic functions, and their redox states respond differently to changes in energy substrate availability [34]. Cytosolic NAD^+^/NADH reflects glycolysis, whereas mitochondrial NAD^+^/NADH reflects the metabolic balance of the cell in generating adenosine triphosphate through oxidative phosphorylation [38], β-oxidation of fatty acids [39], and ketone metabolism [40]. Because intact mitochondria are impermeable to NADH, NADH equivalents are transferred from the cytosol into mitochondria via the α-glycerophosphate and malate-aspartate shuttles. Fasting or KD may limit shuttle capacity, potentially by decreasing shuttle components, such as α-ketoglutarate [41]. In the final step of alcohol metabolism, mitochondrial ALDH requires NAD⁺ to convert acetaldehyde to acetate. We also observed sex- and diet-specific correlation patterns among blood ketone and glucose levels, body weight, liver ADH1, lactate and pyruvate levels, and lactate/pyruvate and [NAD⁺]/[NADH] ratios, indicating context-dependent relationships among metabolic variables. However, a strong inverse correlation between lactate/pyruvate and [NAD + ]/[NADH] ratios was consistent across groups, reflecting complementary aspects of cellular redox state. Further studies are needed to investigate how a KD influences mitochondrial [NAD^+^]/[NADH] levels and the downstream consequences for alcohol metabolism [42].

We previously showed that a history of a KD reduced alcohol self-administration in a rat model of alcohol dependence [8]. In the present study, we investigated the effects of concurrent KD exposure and alcohol vapor exposure in male and female rats using a “clamped” procedure. KD-fed rats were exposed to lower concentrations of alcohol vapor than chow-fed rats in a daily 14 h on (intoxication)/10 h off (withdrawal) schedule to account for diet-related differences in alcohol metabolism. This approach resulted in equivalent BALs between KD- and chow-fed rats during alcohol vapor exposure. Alcohol-dependent male KD-fed rats exhibited a reduced alcohol self-administration beginning on day 5 of testing compared with male chow-fed rats. This reduction persisted after controlling for group differences in body weight and expressed alcohol intake in g/kg of bodyweight. We did not observe sex differences in alcohol intake, consistent with several reports using operant models [21, 43]. Although BALs were not measured following operant sessions in the present study, previous studies showed that the number of lever presses that were made by rats on the alcohol-associated lever to receive an alcohol delivery positively correlated with BALs that were taken immediately following an operant session [44]. In other words, this operant procedure measures appetitive response to alcohol, which increases when rats are made dependent on alcohol by daily, intermittent exposure to alcohol vapor. When considering the reduction of alcohol metabolism in KD-exposed rats, lower levels of alcohol self-administration in male KD-fed rats could produce similar BALs compared with male chow-fed rats. In females, we did not observe group differences in alcohol self-administration, which could be due to differences in the length of alcohol vapor exposure and BALs. Nevertheless, the same alcohol intake in KD-fed and chow-fed female rats could lead to higher BALs in female KD-fed rats compared with female chow-fed rats due to reduced alcohol metabolism. Mackowiak et al. showed that male and female ADH1 knockout mice exhibited higher levels of alcohol in blood, the liver, and the cerebellum and a reduction of binge-like alcohol drinking [32]. However, a KD induced changes in hepatic ADH1, enzymes, and cofactors in both males and females but reduced alcohol consumption exclusively in male KD-fed rats. The female data suggest that drinking may also be modulated independently from alcohol metabolism. Reductions of alcohol drinking independent of alcohol metabolism could involve KD-induced reductions of craving, neuroinflammation, and alcohol withdrawal [2, 8]. Moreover, body weight was significantly lower in male KD-fed rats, whereas no effect was observed in females, suggesting sex-specific metabolic adaptations, which may have contributed to the reduction of drinking that was observed only in male rats. A KD may reduce alcohol consumption through multiple mechanisms, including alterations of alcohol metabolism, shifts in brain energy substrates via fatty acid oxidation, the modulation of glutamate and γ-aminobutyric acid neurotransmission, effects on K⁺ and Ca²⁺ channels, and changes in hormonal signaling pathways that are involved in feeding and addiction (e.g., glucocorticoids, mineralocorticoids, ghrelin, leptin, and glucagon-like peptide 1) [2, 19, 45].

Note that only alcohol-dependent rats were tested in the present study. Blanco-Gandia et al. demonstrated that a KD decreased alcohol intake in nondependent mice [15], and we previously found that a history of KD did not alter alcohol self-administration in nondependent rats [8]. The effect of a concurrent KD on alcohol drinking in nondependent male and female rats remains to be tested. Furthermore, although a KD affected crossovers during operant sessions in both sexes, it did not affect operant responding for water, suggesting that a KD did not impair the rat’s ability to perform an operant response to obtain alcohol.

Our findings suggest sex differences in the effects of the KD. We observed KD-associated reductions in body weight and blood glucose levels in male, but not female, rats; a pattern consistent with previous reports [46, 47]. Similarly, clinical studies in individuals with obesity have shown that adherence to a KD reduces body weight in both sexes, with a more pronounced effect in men [48–50], and reduces HbA1c specifically in men [48]. Another preclinical study demonstrated that a 3-week KD did not alter body weight in male mice, but induced weight gain in female mice, and increased markers of cellular senescence and oxidative stress in males only [51]. The KD has also shown efficacy in improving neuropsychiatric outcomes, although these actions appear to be sex specific. KD significantly attenuated neuroimmune markers in the prefrontal cortex and hippocampus and reduced behavioral measures of anxiincety in male, but not female, rats [52]. In the present study, we found that KD alters brain energetics; however, PET [^18^F]FDG scans were conducted in male rats only. Because sex differences in KD-associated changes in blood glucose levels were observed herein and may have implications for brain glucose metabolism [53–55], the absence of PET [^18^F]FDG scans in females is a limitation that prevented direct comparison across sexes. Nevertheless, prior human studies have found no apparent sex differences in reductions in brain glucose metabolism with a KD [26] or with exogenous ketone supplementation [56].

We also observed decreased alcohol consumption following a KD in male and not female rats. This sex-specific reduction may reflect differential effects of KD on brain energetics and/or other brain processes, a possibility that warrants further investigation. Alcohol consumption shifts brain energetics away from glucose toward greater reliance on acetate, a metabolite of alcohol [4, 5, 29]. In individuals with AUD, it has been hypothesized that a deprivation of acetate during abstinence creates a state of energy deficiency that contributes to alcohol craving and relapse [2, 4, 8]. The therapeutic benefit of a KD may arise from ketone bodies, which are converted to acetyl-CoA and enter the tricarboxylic acid cycle to supply energy, similarly to acetate. Thus, it is possible that a KD induces sex-dependent shifts in brain energetics, paralleling the observed sex differences in blood glucose, and contributes to the sex-specific effects on alcohol consumption.

Emerging evidence suggests the clinical efficacy of KD as an adjunctive therapy for AUD. However, our study findings highlight a potential risk of heightened intoxication and increased vulnerability to alcohol-related harms during relapse in individuals with AUD who are on a KD. Here, we showed that chronic exposure to a KD reduced hepatic alcohol metabolism in both sexes, resulting in higher BALs following alcohol administration. Our study also corroborates prior work demonstrating that a KD reduces alcohol self-administration, but only in male alcohol-dependent rats, and that this reduction appears independent of KD’s effects on alcohol metabolism [8]. Collectively, these findings underscore important sex differences in alcohol drinking responses and potential treatment outcomes associated with the KD.

## Supplementary information

Supplement to A Ketogenic Diet Reduces Hepatic Alcohol Metabolism and Alcohol Consumption in Rats (

## Data Availability

Data are available from the corresponding author upon reasonable request.

## References

[1] Shield K, Manthey J, Rylett M, Probst C, Wettlaufer A, Parry CDH, et al. National, regional, and global burdens of disease from 2000 to 2016 attributable to alcohol use: a comparative risk assessment study. Lancet Public Health. 2020;5:e51–e61. 31910980 10.1016/S2468-2667(19)30231-2

[2] Mahajan VR, Elvig SK, Vendruscolo LF, Koob GF, Darcey VL, King MT, et al. Nutritional ketosis as a potential treatment for alcohol use disorder. Front Psychiatry. 2021;12:781668. 34916977 10.3389/fpsyt.2021.781668 PMC8670944

[3] Courchesne-Loyer A, Croteau E, Castellano CA, St-Pierre V, Hennebelle M, Cunnane SC. Inverse relationship between brain glucose and ketone metabolism in adults during short-term moderate dietary ketosis: a dual tracer quantitative positron emission tomography study. J Cereb Blood Flow Metab. 2017;37:2485–93. 27629100 10.1177/0271678X16669366 PMC5531346

[4] Volkow ND, Kim SW, Wang GJ, Alexoff D, Logan J, Muench L, et al. Acute alcohol intoxication decreases glucose metabolism but increases acetate uptake in the human brain. Neuroimage. 2013;64:277–83. 22947541 10.1016/j.neuroimage.2012.08.057 PMC3508320

[5] Volkow ND, Wiers CE, Shokri-Kojori E, Tomasi D, Wang GJ, Baler R. Neurochemical and metabolic effects of acute and chronic alcohol in the human brain: studies with positron emission tomography. Neuropharmacology. 2017;122:175–88. 28108358 10.1016/j.neuropharm.2017.01.012

[6] Tonetto S, Weikop P, Thomsen M. Nutritional ketosis as treatment for alcohol withdrawal symptoms in female C57BL/6J mice. Sci Rep. 2024;14:5092. 38429369 10.1038/s41598-024-55310-3 PMC10907582

[7] Dencker D, Molander A, Thomsen M, Schlumberger C, Wortwein G, Weikop P, et al. Ketogenic diet suppresses alcohol withdrawal syndrome in rats. Alcohol Clin Exp Res. 2018;42:270–77. 29160944 10.1111/acer.13560

[8] Wiers CE, Vendruscolo LF, van der Veen JW, Manza P, Shokri-Kojori E, Kroll DS, et al. Ketogenic diet reduces alcohol withdrawal symptoms in humans and alcohol intake in rodents. Sci Adv. 2021;7:eabf6780. 10.1126/sciadv.abf6780 PMC8034849 33837086

[9] Quertemont E. Genetic polymorphism in ethanol metabolism: acetaldehyde contribution to alcohol abuse and alcoholism. Mol Psychiatry. 2004;9:570–81. 15164086 10.1038/sj.mp.4001497

[10] Cederbaum AI. Alcohol metabolism. Clin Liver Dis. 2012;16:667–85. 23101976 10.1016/j.cld.2012.08.002 PMC3484320

[11] Bosron WF, Crabb DW, Housinger TA, Li TK. Effect of fasting on the activity and turnover of rat liver alcohol dehydrogenase. Alcohol Clin Exp Res. 1984;8:196–200. 6375431 10.1111/j.1530-0277.1984.tb05837.x

[12] Lumeng L, Bosron WF, Ting-Kai L. Quantitative correlation of ethanol elimination rates in vivo with liver alcohol dehydrogenase activities in fed, fasted and food-restricted rats. Biochem Pharmacol. 1979;28:1547–51. 475866 10.1016/0006-2952(79)90471-4

[13] Finnigan F, Hammersley R, Millar K. Effects of meal composition on blood alcohol level, psychomotor performance and subjective state after ingestion of alcohol. Appetite. 1998;31:361–75. 9920688 10.1006/appe.1998.0168

[14] Rogers J, Smith J, Starmer GA, Whitfield JB. Differing effects of carbohydrate, fat and protein on the rate of ethanol metabolism. Alcohol Alcohol. 1987;22:345–53. 3426763

[15] Blanco-Gandía MD, Ródenas-González F, Pascual M, Reguilón MD, Guerri C, Miñarro J, et al. Ketogenic diet decreases alcohol intake in adult male mice. Nutrients. 10.3390/nu13072167. 10.3390/nu13072167 PMC8308435 34202492

[16] Torres-Rubio L, Reguilón MD, Mellado S, Pascual M, Rodríguez-Arias M. Effects of ketogenic diet on increased ethanol consumption induced by social stress in female mice. Nutrients. 10.3390/nu16172814. 10.3390/nu16172814 PMC11397041 39275131

[17] Vendruscolo LF, Roberts AJ. Operant alcohol self-administration in dependent rats: focus on the vapor model. Alcohol. 2014;48:277–86. 24290310 10.1016/j.alcohol.2013.08.006 PMC4007394

[18] Pawlosky RJ, Kemper MF, Kashiwaya Y, King MT, Mattson MP, Veech RL. Effects of a dietary ketone ester on hippocampal glycolytic and tricarboxylic acid cycle intermediates and amino acids in a 3xTgAD mouse model of Alzheimer’s disease. J Neurochem. 2017;141:195–207. 28099989 10.1111/jnc.13958 PMC5383517

[19] Bergman C, Kashiwaya Y, Veech RL. The effect of pH and free Mg2+ on ATP linked enzymes and the calculation of Gibbs free energy of ATP hydrolysis. J Phys Chem B. 2010;114:16137–46. 20866109 10.1021/jp105723r

[20] Williamson DH, Lund P, Krebs HA. The redox state of free nicotinamide-adenine dinucleotide in the cytoplasm and mitochondria of rat liver. Biochem J. 1967;103:514–27. 4291787 10.1042/bj1030514 PMC1270436

[21] Priddy BM, Carmack SA, Thomas LC, Vendruscolo JC, Koob GF, Vendruscolo LF. Sex, strain, and estrous cycle influences on alcohol drinking in rats. Pharm Biochem Behav. 2017;152:61–67. 10.1016/j.pbb.2016.08.001 PMC5755698 27498303

[22] Vendruscolo LF, Barbier E, Schlosburg JE, Misra KK, Whitfield TW Jr., Logrip ML, et al. Corticosteroid-dependent plasticity mediates compulsive alcohol drinking in rats. J Neurosci. 2012;32:7563–71. 22649234 10.1523/JNEUROSCI.0069-12.2012 PMC3375621

[23] Yuan X, Wang J, Yang S, Gao M, Cao L, Li X, et al. Effect of the ketogenic diet on glycemic control, insulin resistance, and lipid metabolism in patients with T2DM: a systematic review and meta-analysis. Nutr Diab. 2020;10:38. 10.1038/s41387-020-00142-z PMC7705738 33257645

[24] Luong TV, Pedersen MGB, Abild CB, Lauritsen KM, Kjærulff MLG, Møller N, et al. A 3-week ketogenic diet increases skeletal muscle insulin sensitivity in individuals with obesity: a randomized controlled crossover trial. Diabetes. 2024;73:1631–40. 39052652 10.2337/db24-0162 PMC11417439

[25] LaManna JC, Salem N, Puchowicz M, Erokwu B, Koppaka S, Flask C, et al. Ketones suppress brain glucose consumption. Adv Exp Med Biol. 2009;645:301–6. 19227486 10.1007/978-0-387-85998-9_45 PMC2874681

[26] Bennett OA, Ramsay SC, Malacova E, Bourgeat P, Goodman SJ, Dunn CJ, et al. Regional differences in the reduction in cerebral FDG uptake induced by the ketogenic diet. Eur J Hybrid Imaging. 2022;6:29. 36517647 10.1186/s41824-022-00150-5 PMC9751237

[27] Bentourkia M, Tremblay S, Pifferi F, Rousseau J, Lecomte R, Cunnane S. PET study of 11C-acetoacetate kinetics in rat brain during dietary treatments affecting ketosis. Am J Physiol Endocrinol Metab. 2009;296:E796–E801. 19176356 10.1152/ajpendo.90644.2008

[28] Courchesne-Loyer A, Croteau E, Castellano C-A, St-Pierre V, Hennebelle M, Cunnane SC. Inverse relationship between brain glucose and ketone metabolism in adults during short-term moderate dietary ketosis: a dual tracer quantitative positron emission tomography study. J Cereb Blood Flow Metab. 2017;37:2485–93. 27629100 10.1177/0271678X16669366 PMC5531346

[29] Volkow ND, Wang GJ, Shokri Kojori E, Fowler JS, Benveniste H, Tomasi D. Alcohol decreases baseline brain glucose metabolism more in heavy drinkers than controls but has no effect on stimulation-induced metabolic increases. J Neurosci. 2015;35:3248–55. 25698759 10.1523/JNEUROSCI.4877-14.2015 PMC4331638

[30] Ramchandani VA, Kwo PY, Li TK. Effect of food and food composition on alcohol elimination rates in healthy men and women. J Clin Pharmacol. 2001;41:1345–50. 11762562 10.1177/00912700122012814

[31] Deltour L, Foglio MH, Duester G. Metabolic deficiencies in alcohol dehydrogenase Adh1, Adh3, and Adh4 null mutant mice. Overlapping roles of Adh1 and Adh4 in ethanol clearance and metabolism of retinol to retinoic acid. J Biol Chem. 1999;274:16796–801. 10358022 10.1074/jbc.274.24.16796

[32] Mackowiak B, Haggerty DL, Lehner T, Lin YH, Fu Y, Lu H, et al. Peripheral alcohol metabolism dictates ethanol consumption and drinking microstructure in mice. Alcohol Clin Exp Res. 2025;49:970–84. 10.1111/acer.70036 PMC12097942 40114621

[33] Elamin M, Ruskin DN, Masino SA, Sacchetti P. Ketone-based metabolic therapy: is increased NAD(+) a primary mechanism? Front Mol Neurosci. 2017;10:377. 29184484 10.3389/fnmol.2017.00377 PMC5694488

[34] Hu Q, Wu D, Walker M, Wang P, Tian R, Wang W. Genetically encoded biosensors for evaluating NAD(+)/NADH ratio in cytosolic and mitochondrial compartments. Cell Rep Methods. 2021;1:100116. 10.1016/j.crmeth.2021.100116 PMC8659198 34901920

[35] Xin L, Ipek Ö, Beaumont M, Shevlyakova M, Christinat N, Masoodi M, et al. Nutritional ketosis increases NAD(+)/NADH ratio in healthy human brain: an in vivo study by (31)P-MRS. Front Nutr. 2018;5:62. 30050907 10.3389/fnut.2018.00062 PMC6052097

[36] Venkataramaiah TH, Plapp BV. Formamides mimic aldehydes and inhibit liver alcohol dehydrogenases and ethanol metabolism. J Biol Chem. 2003;278:36699–706. 12855684 10.1074/jbc.M305419200

[37] Wu K, Li J, Zhou X, Zhou F, Tang S, Yi l, et al. NADH and NRH as potential dietary supplements or pharmacological agents for early liver injury caused by acute alcohol exposure. J Funct Foods. 2021;87:104852.

[38] Lautrup S, Sinclair DA, Mattson MP, Fang EF. NAD(+) in brain aging and neurodegenerative disorders. Cell Metab. 2019;30:630–55. 31577933 10.1016/j.cmet.2019.09.001 PMC6787556

[39] Bartlett K, Eaton S. Mitochondrial β-oxidation. Eur J Biochem. 2004;271:462–69. 14728673 10.1046/j.1432-1033.2003.03947.x

[40] Puchalska P, Crawford PA. Multi-dimensional roles of ketone bodies in fuel metabolism, signaling, and therapeutics. Cell Metab. 2017;25:262–84. 28178565 10.1016/j.cmet.2016.12.022 PMC5313038

[41] Yudkoff M, Daikhin Y, Melø TM, Nissim I, Sonnewald U, Nissim I. The ketogenic diet and brain metabolism of amino acids: relationship to the anticonvulsant effect. Annu Rev Nutr. 2007;27:415–30. 17444813 10.1146/annurev.nutr.27.061406.093722 PMC4237068

[42] Zakhari S. Overview: how is alcohol metabolized by the body? Alcohol Res Health. 2006;29:245–54. 17718403 PMC6527027

[43] Tunstall BJ, Vendruscolo LF, Allen–Worthington K. Chapter 26 - Rat models of alcohol use disorder. In: Suckow MA, Hankenson FC, Wilson RP, Foley PL, editors. The laboratory Rat, 3rd ed. Amsterdam: Academic Press; 2020. pp. 967–86.

[44] Gilpin NW, Karanikas CA, Richardson HN. Adolescent binge drinking leads to changes in alcohol drinking, anxiety, and amygdalar corticotropin releasing factor cells in adulthood in male rats. PLoS One. 2012;7:e31466. 22347484 10.1371/journal.pone.0031466 PMC3275622

[45] Loften A, Farokhnia M, Vendruscolo LF, Leggio L. Neuroendocrinology meets addiction: emerging pharmacotherapies on the horizon. J Intern Med. 2025;298:392–423. 10.1111/joim.70021 PMC12522537 40985188

[46] Smolensky I, Zajac-Bakri K, Odermatt TS, Brégère C, Cryan JF, Guzman R, et al. Sex-specific differences in metabolic hormone and adipose tissue dynamics induced by moderate low-carbohydrate and ketogenic diet. Sci Rep. 2023;13:16465. 37777528 10.1038/s41598-023-43587-9 PMC10542803

[47] Zhang Y, Cochran JD, Souvenir RA, Tai W, Xia R, Gladwin BS, et al. Sex differences in ketogenic diet response reveal gonadal hormone interaction with FGF21 in mice. J Endocr Soc. 2025;9:bvaf131. 40917105 10.1210/jendso/bvaf131 PMC12411846

[48] D’Abbondanza M, Ministrini S, Pucci G, Nulli Migliola E, Martorelli E-E, Gandolfo V, et al. Very low-carbohydrate ketogenic diet for the treatment of severe obesity and associated non-alcoholic fatty liver disease: the role of sex differences. Nutrients. 2020;12:2748. 32916989 10.3390/nu12092748 PMC7551320

[49] Muscogiuri G, Verde L, Frias-Toral E, Reytor-González C, Annunziata G, Proganò M, et al. Weight loss, changes in body composition and inflammatory status after a very low-energy ketogenic therapy (VLEKT): does gender matter? J Transl Med. 2024;22:949. 39427162 10.1186/s12967-024-05733-3 PMC11490016

[50] Jiao Y, Chen X, Liu L, Lu Y, Gao M, Wang Q, et al. Sex differences in ketogenic diet: are men more likely than women to lose weight? Front Nutr. 2025;12:1600927. 40535039 10.3389/fnut.2025.1600927 PMC12173872

[51] Wei S-J, Schell J, Qian W, Silguero M, Baseviciene A, Chen WH, et al. Divergent sex-specific effects on a ketogenic diet: Male, but not female, mice exhibit oxidative stress and cellular senescence. Cell Rep. 2025;44:116026. 10.1016/j.celrep.2025.116026 40682777

[52] Kumar M, Bhatt B, Gusain C, Mahajan N, Bishnoi M. Sex-specific effects of ketogenic diet on anxiety-like behavior and neuroimmune response in C57Bl/6J mice. J Nutritional Biochem. 2024;127:109591. 10.1016/j.jnutbio.2024.109591 38311044

[53] Viglianti BL, Wong KK, Wimer SM, Parameswaran A, Nan B, Ky C, et al. Effect of hyperglycemia on brain and liver (18)F-FDG standardized uptake value (FDG SUV) measured by quantitative positron emission tomography (PET) imaging. Biomed Pharmacother. 2017;88:1038–45. 28192877 10.1016/j.biopha.2017.01.166 PMC5553544

[54] Sarikaya I, Albatineh AN, Sarikayaa A. Effect of various blood glucose levels on regional FDG uptake in the brain. Asia Ocean J Nucl Med Biol. 2020;8:46–53. 32064282 10.22038/aojnmb.2019.14418 PMC6994786

[55] Sarikaya I, Sarikaya A, Sharma P. Assessing the effect of various blood glucose levels on ^18^F-FDG activity in the brain, liver, and blood pool. J Nucl Med Technol. 2019;47:313. 31182660 10.2967/jnmt.119.226969

[56] Li X, Young AJ, Shi Z, Byanyima J, Vesslee S, Reddy R, et al. Pharmacokinetic effects of a single dose nutritional ketone ester supplement on brain glucose and ketone metabolism in alcohol use disorder. Psychiatry Res Neuroimaging. 2026; 357:112154. 10.1016/j.pscychresns.2026.112154 PMC12983345 41633023

