# Supplementary material for: A ketogenic diet reduces hepatic alcohol metabolism and alcohol consumption in rats
Source: Neuropsychopharmacology. 2026 Mar 20;51(9):1568–76. doi: 10.1038/s41386-026-02383-5 (PMC13334472; doi:10.1038/s41386-026-02383-5)
Supplement: Supplementary file 1 — Supplement to A Ketogenic Diet Reduces Hepatic Alcohol Metabolism and Alcohol Consumption in Rats ( [file 41386_2026_2383_MOESM1_ESM.docx]

**A Ketogenic Diet Reduces Hepatic Alcohol Metabolism and Alcohol Consumption in Rats**

Sophie K. Elvig,^1, 2, a^ Adrienne McGinn,^1, a^ Xinyi Li,^3^ Janaina C.M. Vendruscolo,^1^ Juan L. Gomez,^4^ Robert Pawlosky,^5^ Bryan Mackowiak,^6^ Luis Gonzalez,^1^ M. Todd King,^5^ Michael Michaelides,^4^ Bin Gao,^6^ Nora D. Volkow,^7^ George F. Koob,^1^ Corinde E. Wiers,^3,^ *^,^ ^b^ Leandro F. Vendruscolo^8, 9^ *^, b^

**Supplemental Figure S1. Body weight in rats maintained on a ketogenic diet (KD) or chow diet for 8 weeks.** Male KD-fed rats weighted less than male chow-fed rats, whereas no differences were found in female rats. **p* < 0.05, ***p* < 0.01, *****p* < 0.0001, difference between chow and KD. *n* = 8 per group. Diet effect: *F*_1,28_ = 22.87, *p* < 0.0001; sex effect: *F*_1,28_ = 506.96, *p* < 0.0001; diet × sex interaction: *F*_1,28_ = 5.35, *p* = 0.0283; time effect: *F*_4,112_ = 132.31, *p* < 0.0001; diet × time interaction: *F*_4,112_ = 9.31, *p* < 0.0001; sex × time interaction: *F*_4,112_ = 13.33, *p* < 0.0001; diet × sex × time interaction: *F*_4,112_ = 12.81, *p* < 0.0001. *Post hoc* comparisons of the diet × sex × time interaction indicated that male KD-fed rats weighed less than male chow-fed rats at week 1 (*p* = 0.0267), week 2 (*p* = 0.0036), and weeks 4-8 (*p* < 0.0001). No group differences were found in females.

**Table S1.** Pearson correlation matrix of variables in chow-fed male rats.

|  | **Glucose** | **Ketones** | **Body Weight** | **ADH1** | **Lactate** | **Pyruvate** | **Lactate/Pyruvate** |
| --- | --- | --- | --- | --- | --- | --- | --- |
| **Ketones** | -0.5684 |  |  |  |  |  |  |
| **Body Weight** | 0.2945 | -0.1895 |  |  |  |  |  |
| **ADH1** | 0.0033 | -0.0284 | **-0.7921*** |  |  |  |  |
| **Lactate** | 0.2805 | -0.2573 | 0.4882 | **-0.7902*** |  |  |  |
| **Pyruvate** | -0.3500 | 0.0608 | 0.2627 | **-0.7473*** | **0.7499*** |  |  |
| **Lactate/Pyruvate** | **0.8318**** | -0.2275 | 0.0974 | 0.1180 | 0.1682 | -0.4948 |  |
| **NAD^+^/NADH** | **-0.8861**** | 0.3684 | -0.2695 | -0.0070 | -0.2671 | 0.4269 | **-0.9459****** |

ADH1: Alcohol dehydrogenase. **p* < 0.05, ***p* < 0.01, *****p* < 0.0001. *n* = 8 for all variables, except for ADH1 (*n* = 6).

**Table S2.** Pearson correlation matrix of variables in KD-fed male rats.

|  | **Glucose** | **Ketones** | **Body Weight** | **ADH1** | **Lactate** | **Pyruvate** | **Lactate/Pyruvate** |
| --- | --- | --- | --- | --- | --- | --- | --- |
| **Ketones** | **-0.8165*** |  |  |  |  |  |  |
| **Body Weight** | 0.6985 | -0.4266 |  |  |  |  |  |
| **ADH1** | -0.0934 | -0.0746 | 0.0402 |  |  |  |  |
| **Lactate** | 0.1748 | -0.0158 | 0.3108 | 0.2619 |  |  |  |
| **Pyruvate** | 0.4070 | -0.1129 | 0.5004 | 0.1332 | **0.9356***** |  |  |
| **Lactate/Pyruvate** | -0.5036 | 0.0673 | -0.3341 | 0.3703 | -0.0013 | -0.3226 |  |
| **NAD^+^/NADH** | 0.5354 | -0.1254 | 0.3840 | -0.2110 | -0.0053 | 0.3285 | **-0.9869****** |

ADH1: Alcohol dehydrogenase. ****p* < 0.001, *****p* < 0.0001. *n* = 8.

**Table S3.** Pearson correlation matrix of variables in chow-fed female rats.

|  | **Glucose** | **Ketones** | **Body Weight** | **ADH1** | **Lactate** | **Pyruvate** | **Lactate/Pyruvate** |
| --- | --- | --- | --- | --- | --- | --- | --- |
| **Ketones** | 0.0974 |  |  |  |  |  |  |
| **Body Weight** | -0.2208 | 0.0496 |  |  |  |  |  |
| **ADH1** | -0.0041 | -0.1280 | -0.4984 |  |  |  |  |
| **Lactate** | **0.8306*** | -0.0143 | 0.1223 | -0.1861 |  |  |  |
| **Pyruvate** | 0.5670 | -0.2067 | -0.0483 | 0.0813 | **0.8146*** |  |  |
| **Lactate/Pyruvate** | -0.1282 | 0.1573 | 0.0724 | -0.1815 | -0.3505 | **-0.7893*** |  |
| **NAD^+^/NADH** | 0.1092 | -0.2282 | -0.1012 | 0.3050 | 0.3609 | **0.8164*** | **-0.9805****** |

ADH1: Alcohol dehydrogenase. **p* < 0.05, *****p* < 0.0001. *n* = 8.

**Table S4.** Pearson correlation matrix of variables in KD-fed female rats.

|  | **Glucose** | **Ketones** | **Body Weight** | **ADH1** | **Lactate** | **Pyruvate** | **Lactate/Pyruvate** |
| --- | --- | --- | --- | --- | --- | --- | --- |
| **Ketones** | -0.2721 |  |  |  |  |  |  |
| **Body Weight** | -0.2645 | -0.4720 |  |  |  |  |  |
| **ADH1** | 0.7013 | 0.1059 | -0.0575 |  |  |  |  |
| **Lactate** | 0.5677 | 0.3794 | **-0.7796*** | 0.2809 |  |  |  |
| **Pyruvate** | 0.1920 | 0.1941 | -0.6751 | -0.2137 | 0.6229 |  |  |
| **Lactate/Pyruvate** | 0.3001 | 0.1896 | 0.0392 | 0.5387 | 0.2922 | -0.5485 |  |
| **NAD^+^/NADH** | -0.3524 | -0.2283 | -0.0085 | -0.5523 | -0.2973 | 0.5533 | **-0.9660****** |

ADH1: Alcohol dehydrogenase. **p* < 0.05, *****p* < 0.0001. *n* = 8.

**Supplemental Figure S2. Blood alcohol levels in rats maintained on a ketogenic diet (KD) or chow diet for 7-9 weeks.** Males (*n* = 10 per group): diet effect: *F*_1,18_ = 0.74, *p* = 0.4007; time effect: *F*_6,108_ = 4.24, *p* = 0.0007; diet × time interaction: *F*_6,108_ = 3.80, *p* = 0.0018. *Post hoc* comparisons of the diet × time interaction did not indicate significant group differences when comparing each timepoint. Only a few female rats were sampled in weeks 1, 2, and 7 (*n* = 3-4 per timepoint). At week 9, we measured BALs in 8 females per group. Student’s *t*-test did not show a significant group effect (*t*_14_ = 0.60, *p* = 0.1368).

**Supplemental Figure S3. Inactive lever pressing and crossovers in rats maintained on a ketogenic diet (KD).** **(A, B)** Male rats performed more inactive lever presses than female regardless of diet. *n* = 10 for males, 15-16 for females. Diet effect: *F*_1,47_ = 0.33, *p* = 0.5683; sex effect: *F*_1,47_ = 36.60, *p* < 0.0001; diet × sex interaction: *F*_1,47_ = 0.02, *p* = 0.9011; time effect: *F*_9,423_ = 3.33, *p* = 0.0006; diet × time interaction: *F*_9,423_ = 0.58, *p* = 0.8173; sex × time interaction: *F*_9,423_ = 4.09, *p* < 0.0001; diet × sex × time interaction: *F*_9,423_ = 0.76, *p* = 0.6494. **(C, D)** Rats on the Chow diet performed more crossovers than rats on the KD regardless of sex. *n* = 7-9 for males, *n* = 14-15 for females. Note that the discrepancy in sample sizes for inactive lever presses and crossovers was due the fact that not all chambers were equipped with photocell beams. Diet effect: *F*_1,39_ = 4.57, *p* = 0.0388; sex effect: *F*_1,39_ = 0.01, *p* = 0.9923; diet × sex interaction: *F*_1,39_ = 0.05, *p* = 0.8313; time effect: *F*_9,351_ = 2.98, *p* = 0.0012; diet × time interaction: *F*_9,351_ = 0.62, *p* = 0.7747; sex × time interaction: *F*_9,351_ = 3.06, *p* = 0.0015; diet × sex × time interaction: *F*_9,351_ = 1.09, *p* = 0.3682.
